# A Lossless Scalar Calibration Algorithm Used for Tri-Axial Magnetometer Cross Array and Its Effectiveness Validation

**DOI:** 10.3390/s25072164

**Published:** 2025-03-28

**Authors:** Lihua Wu, Yu Huang, Xintong Chen

**Affiliations:** 1 College of Physics and Optoelectronic Engineering, Harbin Engineering University, Harbin 150001, China; wulihua@hrbeu.edu.cn (L.W.); chen.xintong@hrbeu.edu.cn (X.C.); 2 National Key Laboratory of Underwater Acoustic Technology, Harbin Engineering University, Harbin 150001, China

**Keywords:** tri-axial magnetometer cross array, error, lossless scalar calibration, magnetic gradient tensor, effectiveness validation

## Abstract

The accuracy of a magnetic gradient tensor (MGT) measured by tri-axial magnetometer cross arrays (TAMCAs) is compromised by inherent errors in individual tri-axial magnetometers (TAMs) and inter-sensor misalignment angles (MAs), both of which degrade the resultant MGT data quality. This paper proposes a novel lossless scalar calibration algorithm that eliminates mathematical approximations while tracking the fluctuation of the reference magnetic intensity (MI). The calibration algorithm is developed to improve TAMCAs’ measurement precision; however it is difficult to provide a completely accurate MGT by experiments. Therefore, we have designed a kind of validation experiment based on a constrained Euler localization to demonstrate the effectiveness of the calibration algorithm. The fundamental principles of the proposed lossless scalar calibration methodology are systematically presented, accompanied by a numerical analysis of relative errors calibrating TAMCA parameters. Key influencing factors are carefully investigated, including the TAM noise level quantified by standard deviation (STD), calibration dataset size, and STD of reference MI fluctuations. In the experiments, to validate the effectiveness of calibrating TAMCAs composed of four fluxgate TAMs (FTAMs), we measured the true geo-MI using a proton magnetometer and regarded an energized circular coil as the alternating current (AC) magnetic source of the constrained Euler localization, respectively. The results indicated that the lossless scalar calibration algorithm significantly improves the measurement accuracy of the geo-MI of the calibration site and MGT of the energized coil.

## 1. Introduction

As the spatial derivative tensor of magnetic vectors, MGT inherently contains five independent components, demonstrating superior spatial resolution and enhanced signal discrimination capability compared to conventional analytical signals and vector measurements [1,2]. The form of the tensor enables the comprehensive reconstruction of magnetic source architectures while simultaneously suppressing geomagnetic noise and eliminating reliance on fixed base stations. Crucially, MGT permits direct determination of magnetization parameters through its rotationally invariant properties, requiring neither prior knowledge of source geometry nor positional information, and can show more effective capability in shallow magnetic anomaly detection over the total field measurement [3]. Unlike magnetic vector measurements vulnerable to attitude-induced errors in the presence of geomagnetic fields, MGT maintains operational stability and exhibits heightened sensitivity to magnetic anomaly signals, owing to its inherent insensitivity to both geomagnetic diurnal variations and spatial orientation uncertainties [4,5]. These characteristics render MGT ideally suited for mobile deployment platforms in challenging environments where traditional magnetic prospecting fails, including low-latitude regions and rugged terrains. Specific applications span concealed mineral exploration, unexploded ordnance localization, and marine detection systems.

The limitations inherent in sensor manufacturing processes and system installation accuracy introduce MAs between different TAMs, in addition instrumental errors within individual TAMs, collectively degrading the data quality of MGT systems and necessitating calibration procedures to mitigate such discrepancies. TAM calibration methods are broadly categorized into vector-based and scalar-based approaches. The vector calibration method employs high-precision magnetic vectors as reference fields to calibrate TAMs, typically requiring magnetically shielded environments or active coil compensation system to suppress external magnetic interference, thereby imposing stringent application constraints. While this method is particularly suitable for characterizing TAM frequency responses [6], its implementation complexity limits practical applicability. In contrast, scalar calibration methods utilize high-precision MIs as reference standards [7,8,9,10], with constrained optimization-based techniques further eliminating the need for scalar magnetometer measurements to achieve cost-effective and simplified implementations [11]. However, a critical limitation of the scalar calibration method lies in the assumption of invariant MI outputs during calibration, which fails to track fluctuations in the reference magnetic field, potentially compromising calibration accuracy under real-world conditions.

The calibration of MAs in MGT systems relies on aligning the measurement benchmarks of TAMs to a unified coordinate system, either defined by a single TAM or the platform itself. In principle, a non-ideal MGT system in a uniform magnetic field produces outputs dependent on the field’s uniformity, as the theoretical magnetic gradient in such regions is zero. This property enables calibration by rotating the MGT system within a uniform field and applying least squares or linear constrained least squares fitting to synchronize MGT-measured data with reference magnetic vectors [12]. For online calibration, Ref. [13] introduced a generalized least squares algorithm tailored to towed airborne fluxgate MGT systems, leveraging static error models, fluxgate dynamic characteristics, and the near-zero geo-MGT at high altitudes, validated via helicopter flight tests. However, real-world geomagnetic environments rarely satisfy uniform-field conditions, driving researchers to explore tensor invariant-based calibration strategies. Ref. [14] proposed calibrating MGT systems using two tensor invariants as constraints, analogous to scalar TAM calibration. Ref. [15] further integrated tensor components and invariants into an objective function, employing an improved differential evolution algorithm to simultaneously correct TAM instrumental errors and MAs in a single step, demonstrating enhanced noise resistance over conventional least squares methods. Additionally, an indirect calibration approach utilizing the generalized Hilbert transform was developed for airborne systems, bypassing the need for direct reference fields by leveraging high-precision total field gradiometer data as a processed calibration benchmark [16]. Faced with the challenges brought by non-uniform fields and dynamic operating environments, these advancements have collectively improved the accuracy of MGT systems under practical conditions.

Ref. [17] applies both implemented and optimized calibration approaches to full MGT systems utilizing superconducting quantum interference devices (SQUIDs), emphasizing rapid data processing, yet omitting explicit solutions for calibrating MAs. For tetrahedral MGT systems, Ref. [18] introduces a calibration algorithm based on constraint relationships among the nine components of the MGT, improving accuracy through inherent physical interdependencies. However, these constraints cannot directly extend to TAMCAs measuring only five independent tensor components. Ref. [19] proposes a linear calibration method for TAMCAs relying solely on vertical magnetic field constraints, which proves insufficient for comprehensive MA correction. Ref. [20] addresses this by constructing a nonlinear correction model within an artificial platform reference frame and employing the Levenberg–Marquardt algorithm for parameter fitting; however, the coupling between MAs and orthogonal error angles (OEAs) introduces interdependencies that challenge solution independence and accuracy. Ref. [21] enhances TAMCA calibration precision by adopting total least squares for ellipsoidal model parameter fitting, outperforming conventional least squares, yet fails to provide explicit sensor error parameter calculations. Meanwhile, MGT measurements are typically conducted via differential approximation, rotation modulation [22], or string vibration methods [23], with the differential approximation method favored for its simplicity and ability to capture full tensor information. For this method, centrally symmetric TAMCAs are preferred due to their minimized principle-based measurement errors [24]. Collectively, the calibration of MGT systems involves diverse methodologies with varying scopes and limitations.

This paper proposes a lossless scalar calibration algorithm for TAMCAs that eliminates mathematical approximations while dynamically tracking fluctuations in the reference MI. The algorithm derives an initial solution by linearizing the ellipsoid model equation, thereby accelerating computational efficiency. The structure of the paper is organized as follows. Section 2 details the principles of the proposed lossless scalar calibration algorithm for TAMCAs. Section 3 outlines validation strategies to assess the algorithm’s effectiveness. In Section 4, numerical simulations analyze calibration accuracy under varying noise conditions, including dependencies on the STDs of TAM and scalar magnetometer noise, as well as calibration dataset size. Experimental validation is further conducted through geomagnetic field measurements and constrained Euler localization of an energized circular coil. Finally, Section 5 synthesizes key conclusions.

## 2. Calibration Methodology

### 2.1. Error Model of the TAMCA

**Figure 1:**
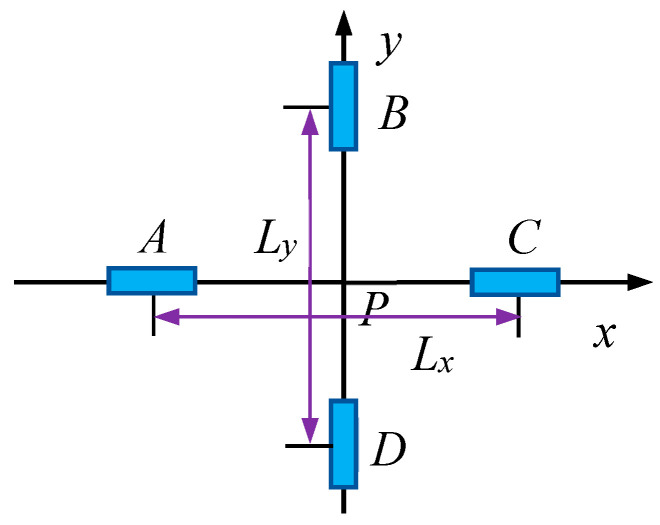
Schematic configuration of the TAMCA.

The TAMCA comprises four TAMs positioned at vertices A, B, C, and D, labeled as nodes 1, 2, 3, and 4, respectively, as shown in Figure 1. Benefitting from the cross-shaped symmetric configuration of the array, the measured magnetic vector and MGT can effectively approximate to their true values at the geometric center P of the array. The baseline lengths of the array, defined as the distances *L*_x_ and *L*_y_ between the paired TAMs along the *x*- and *y*-axes, characterize the spatial resolution of the array in the *x*- and *y*- directions, respectively, served as critical parameters for spatial gradient calculations.

**Figure 2:**
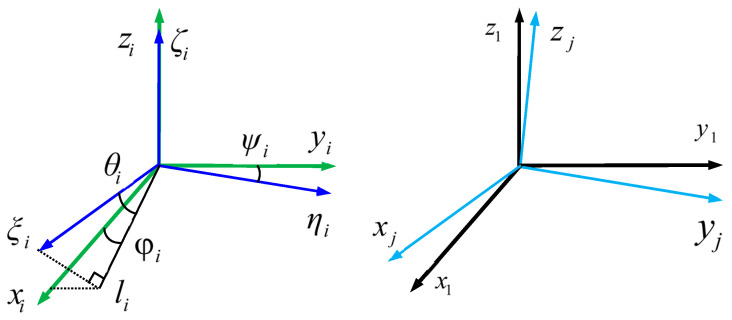
Spatial orientation of sensor’s sensitive axes in the TAMCA.

The measurement errors of the TAMCA stem from two primary sources: (1) intrinsic errors of the four TAMs including scale factor errors (SFEs), OEAs, and biases; and (2) MAs induced during system assembly. Calibrating the TAMCA as an integrated system, rather than individually calibrating each TAM, mitigates MAs caused by installation misalignments. As illustrated in Figure 2, the sensor coordinate system ($\xi_{i}\eta_{i}\zeta_{i}$, i=1,2,3,4) and reference coordinate system ($x_{i}y_{i}z_{i}$) are defined such that their *z*-axes ($\zeta_{i}$ and $z_{i}$) are aligned. The OEAs of the *i*-th TAM are characterized by angles $\theta_{i}$, $\varphi_{i}$, and $\psi_{i}$, where $\psi_{i}$ represents the angle between the $\eta_{i}$-axis and $y_{i}$-axis, $\theta_{i}$ denotes the angle between the $\xi_{i}$-axis and its projection onto the $x_{i}y_{i}$-plane, and $\varphi_{i}$ defines the angle between the projected $l_{i}$-axis and the $x_{i}$-axis. The MAs between the sensor coordinate system $x_{j}y_{j}z_{j}$ (for the *j*-th TAM, j=2,3,4) and the reference system $x_{1}y_{1}z_{1}$ are further quantified by the angles $\gamma_{j}$, $\beta_{j}$, and $\alpha_{j}$ of right-handed sequential rotation (z-y-x), encapsulating the spatial misalignment inherent to the array configuration.

$B_{i}$ represents the true magnetic vector at the point of the *i*-th TAM in the coordinate system $x_{1}y_{1}z_{1}$, and $B_{i}^{\prime }$ is the expression of $B_{i}$ in the coordinate system $x_{i}y_{i}z_{i}$, then

(1) $$B_{i}^{\prime }=\Lambda_{i}^{-1}B_{i}$$

where $\Lambda_{1}$ is an identity matrix with three ranks; namely, $B_{1}^{\prime }=B_{1}$, $\Lambda_{j}=R_{x}(\alpha_{j})R_{y}(\beta_{j})R_{z}(\gamma_{j})$, $R_{x}$, $R_{y}$, and $R_{z}$ represent the rotation matrices around the *x*-axis, *y*-axis, and *z*-axis, respectively.

A uniform magnetic field is selected to calibrate the TAMCA, so the magnetic vectors $B_{i}$ are all the same. The measurement output ${\tilde{B}}_{i}$ of the *i*-th TAM is as follows [21]:

(2) $${\tilde{B}}_{i}=D_{i}B_{i}^{\prime }+B_{0i}+n_{i}$$

(3) $$D_{i}=\left[\begin{array}{ccc} S_{xi}\cos\theta_{i}\cos\varphi_{i} & S_{xi}\cos\theta_{i}\sin\varphi_{i} & S_{xi}\sin\theta_{i}\\ 0 & S_{yi}\cos\psi_{i} & S_{yj}\sin\psi_{i}\\ 0 & 0 & S_{zi} \end{array}\right]$$

where, $D_{i}$, $B_{0i}$, and $n_{i}$ are the measurement matrix, bias, and noise of the *i*-th TAM, and $S_{xi}$, $S_{yi}$, and $S_{zi}$ are the scalar factors (SFs) of the three sensitive axes, respectively. $\delta S_{xi}=S_{xi}-1$, $\delta S_{yi}=S_{yi}-1$, $\delta S_{zi}=S_{zi}-1$ are, respectively, the SFEs. We can obtain from Equations (1) and (2) that

(4) $$B_{i}^{\prime }=\Lambda_{i}^{-1}B_{i}=\Omega_{i}({\tilde{B}}_{i}-B_{0i}-n_{i})$$

where $\Omega_{i}$ is the inversed matrix of $D_{i}$.

Non-zero elements of $\Omega_{i}$ are, respectively, $\Omega_{i}(1,2)=p_{2i}=-\tan\varphi_{i}\sec\psi_{i}/S_{yi}$, $\Omega_{i}(2,3)=p_{5i}=-\tan\psi_{i}/S_{zi}$, $\Omega_{i}(2,2)=p_{4i}=\sec\psi_{i}/S_{yi}$, $\Omega_{i}(1,1)=p_{1i}=\sec\theta_{i}\sec\varphi_{i}/S_{xi}$, $\Omega_{i}(3,3)=p_{6i}=S_{zi}^{-1}$, $\Omega_{i}(1,3)=p_{3i}=\sec\theta_{i}\sec\varphi_{i}\sec\psi_{i}(\cos\theta_{i}\sin\varphi_{i}\sin\psi_{i}-\sin\theta_{i}\cos\psi_{i})/S_{zi}$. Given $S_{xi}$, $S_{yi}$, and $S_{zi}$ approximate unity, while the small angles $\theta_{i}$, $\varphi_{i}$, and $\psi_{i}$ induce non-zero elements of $\Omega_{i}$ constrained to first-order contributions, the parameter identification accuracy is significantly enhanced. The expression of calculating the SFs and OEAs from $p_{ki},k=1,2,\cdots ,6$ is as follows:

(5) $$\left\{\begin{array}{l} S_{zi}=1/p_{6i},\psi_{i}=-\arctan(S_{zi}p_{5i})\\ S_{yi}=\sec\psi_{i}/p_{4i},\varphi_{i}=-\arctan(S_{yi}p_{2i}\cos\psi_{i})\\ \theta_{i}=\arctan(\sin\varphi_{i}\tan\psi_{i}-S_{zi}p_{3i}\cos\varphi_{i}),S_{xi}=\sec\theta_{i}\sec\varphi_{i}/p_{1i} \end{array}\right.$$

### 2.2. Principal of Lossless Scalar Calibration Algorithm

The ellipsoidal equation obtained from Equation (2) is as follows:

(6) $${\tilde{B}}_{i}^{T}A_{i}{\tilde{B}}_{i}-2B_{0i}^{T}A_{i}{\tilde{B}}_{i}+B_{0i}^{T}A_{i}B_{0i}=1$$

where

(7) $$B_{0i}=-A_{i}^{-1}{\left[r_{i}s_{i}t_{i}\right]}^{T}$$

$\left||B_{i}|\right|$ is the reference MI. Generally, $A_{i}=||B_{i}|{|}^{-1}\Omega_{i}^{T}\Omega_{i}$ is a symmetric positive definite matrix that can accurately track the fluctuations of $\left||B_{i}|\right|$, and $A_{i}$ is defined as follows:

(8) $$A_{i}=\left[\begin{array}{ccc} a_{i} & d_{i} & e_{i}\\ d_{i} & b_{i} & f_{i}\\ e_{i} & f_{i} & c_{i} \end{array}\right]$$

Let $u_{i}={\left[a_{i},b_{i},c_{i},d_{i},e_{i},f_{i},r_{i},s_{i},t_{i}\right]}^{T}$ and we can obtain the least square solution $u_{0i}$ as expressed by Equation (9) through linearizing Equation (6).

(9) $$u_{0i}={\left[K_{i}^{T}K_{i}\right]}^{-1}K_{i}^{T}1_{N\times 1}$$

(10) $$K_{i}=\left[\begin{array}{ccccccccc} {\tilde{B}}_{xi1}^{2} & {\tilde{B}}_{yi1}^{2} & {\tilde{B}}_{zi1}^{2} & 2{\tilde{B}}_{xi1}{\tilde{B}}_{yi1} & 2{\tilde{B}}_{xi1}{\tilde{B}}_{zi1} & 2{\tilde{B}}_{yi1}{\tilde{B}}_{zi1} & 2{\tilde{B}}_{xi1} & 2{\tilde{B}}_{yi1} & 2{\tilde{B}}_{zi1}\\ {\tilde{B}}_{xi2}^{2} & {\tilde{B}}_{yi2}^{2} & {\tilde{B}}_{zi2}^{2} & 2{\tilde{B}}_{xi2}{\tilde{B}}_{yi2} & 2{\tilde{B}}_{xi2}{\tilde{B}}_{zi2} & 2{\tilde{B}}_{yi2}{\tilde{B}}_{zi2} & 2{\tilde{B}}_{xi2} & 2{\tilde{B}}_{yi2} & 2{\tilde{B}}_{zi2}\\ \vdots & \vdots & \vdots & \vdots & \vdots & \vdots & \vdots & \vdots & \vdots \\ {\tilde{B}}_{xiN}^{2} & {\tilde{B}}_{yiN}^{2} & {\tilde{B}}_{ziN}^{2} & 2{\tilde{B}}_{xiN}{\tilde{B}}_{yiN} & 2{\tilde{B}}_{xiN}{\tilde{B}}_{ziN} & 2{\tilde{B}}_{yiN}{\tilde{B}}_{ziN} & 2{\tilde{B}}_{xiN} & 2{\tilde{B}}_{yiN} & 2{\tilde{B}}_{ziN} \end{array}\right]$$

What is more, the nonlinear equation system expressed by Equation (6) can be transformed into a constrained optimization problem denoted by Equation (11).

(11) $$\left\{\begin{array}{l} \min f_{i}(u_{i})\\ s.t.u_{iL}<u_{i}<u_{iH} \end{array}\right.$$

where $f_{i}(u_{i})=\sum_{n=1}^{N}y_{i}^{2}(n)$ and N is the calibration dataset size.

(12) $$\begin{array}{ll} y_{i}= & {\tilde{B}}_{xi}^{2}u_{1i}+{\tilde{B}}_{yi}^{2}u_{2i}+{\tilde{B}}_{zi}^{2}u_{3i}+2({\tilde{B}}_{xi}{\tilde{B}}_{yi}u_{4i}+{\tilde{B}}_{xi}{\tilde{B}}_{zi}u_{5i}+{\tilde{B}}_{yi}{\tilde{B}}_{zi}u_{6i}-{\tilde{B}}_{xi}u_{1i}u_{7i}\\ & -{\tilde{B}}_{yi}u_{2i}u_{8i}-{\tilde{B}}_{zi}u_{3i}u_{9i}-{\tilde{B}}_{xi}u_{4i}u_{8i}-{\tilde{B}}_{yi}u_{4i}u_{7i}-{\tilde{B}}_{xi}u_{5i}u_{9i}-{\tilde{B}}_{zi}u_{5i}u_{7i}-{\tilde{B}}_{zi}u_{6i}u_{8i}\\ & -{\tilde{B}}_{yi}u_{6i}u_{9i}+u_{4i}u_{7i}u_{8i}+u_{5i}u_{7i}u_{9i}+u_{6i}u_{8i}u_{9i})+u_{1i}u_{7i}^{2}+u_{2i}u_{8i}^{2}+u_{3i}u_{9i}^{2}-1 \end{array}$$

The sequential quadratic programming algorithm is used to solve Equation (11) to obtain $u_{i}$, regarding $u_{0i}$ as the initial solution, and then substitute $u_{i}$ into Equation (7) to calculate out the biases $B_{0i}={\left[B_{0xi},B_{0yi},B_{0zi}\right]}^{T}$. Equation (12) is unrelated to $\left||B_{i}|\right|$ but related to ${\tilde{B}}_{i}$, so the solution of $B_{0i}$ is not related to the noise of scalar magnetometer measuring $\left||B_{i}|\right|$, but to the noise of the TAM.

After obtaining $B_{0i}$, we can derive the following from Equations (1) and (4):

(13) $$\sum_{k=1}^{6}W_{ki}q_{ki}=||B_{i}^{\prime }|{|}^{2}=||B_{i}|{|}^{2}$$

where $q_{1i}=p_{1i}^{2}$, $q_{2i}=p_{2i}^{2}+p_{4i}^{2}$, $q_{3i}=p_{3i}^{2}+p_{5i}^{2}+p_{6i}^{2}$, $q_{4i}=p_{1i}p_{2i}$, $q_{5i}=p_{2i}p_{3i}+p_{4i}p_{5i}$, $q_{6i}=p_{1i}p_{3i}$, $W_{1i}={\left({\tilde{B}}_{xi}-B_{0xi}\right)}^{2}$, $W_{2i}={\left({\tilde{B}}_{yi}-B_{0yi}\right)}^{2}$, $W_{3i}={\left({\tilde{B}}_{zi}-B_{0zi}\right)}^{2}$, $W_{4i}=2({\tilde{B}}_{xi}-B_{0xi})({\tilde{B}}_{yi}-B_{0yi})$, $W_{5i}=2({\tilde{B}}_{yi}-B_{0yi})({\tilde{B}}_{zi}-B_{0zi})$, $W_{6i}=2({\tilde{B}}_{xi}-B_{0xi})({\tilde{B}}_{zi}-B_{0zi})$.

Equation (13) is solved using linear least squares to obtain $q_{ki}$, and then to calculate $p_{ki}$ using $q_{ki}$, while the SFs and OEAs can be calculated out substituting $p_{ki}$ into Equation (5), which is called a nonlinear fitting transformation. As a result, $B_{i}^{\prime }$ can be calculated out from Equation (4) using the obtained biases, SFs, and OEAs. Equation (13) is related to both ${\tilde{B}}_{i}$ and $\left||B_{i}|\right|$; therefore, the calibration results of the SFEs and OEAs may depend on the noises of both the TAM and scalar magnetometer.

The identification of $\alpha_{j}$, $\beta_{j}$, and $\gamma_{j}$ can be regarded as the Procrustes problem, so the MAs are determined using Procrustes analysis on Equation (1), according to $B_{i}^{\prime }$. Procrustes analysis is a method that directly estimates the translation and rotation parameters of coordinate transformation by performing singular value decomposition on the eigen matrix, which greatly simplifies the calculation.

**Figure 3:**
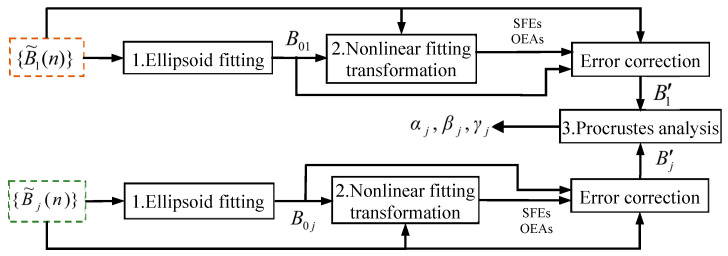
Flowchart of the lossless scalar calibration algorithm for the TAMCA.

In summary, there are no mathematical approximations for a lossless scalar calibration algorithm that can track fluctuations in the reference MI, and it is divided into three steps as shown in Figure 3. The first step is to obtain $B_{0i}$ through solving Equation (11). The second step is to solve Equation (13) using linear least squares to calculate out $q_{ki}$ and $p_{ki}$, and then the SFs and OEAs are all calculated through substituting $p_{ki}$ into Equation (5). In the third step, all of the calculated biases, SFEs, and OEAs are substituted into Equation (4) to obtain $B_{i}^{\prime }$, and then $\alpha_{j}$, $\beta_{j}$, and $\gamma_{j}$ are obtained by Procrustes analysis.

## 3. Calibration Effectiveness Validation Method

### 3.1. Validation Using the Measurement of MI

The root mean square errors (RMSEs) of MI and MGT are often used as the parameters evaluating the performance of the lossless scalar calibration algorithm for the TAMCA. ${\tilde{e}}_{B}$ and ${\hat{e}}_{B}$ are the RMSEs of the measured MI before and after calibrating the TAMCA, which are defined as follows, respectively:

(14) $${\tilde{e}}_{B}=\sqrt{\frac{1}{N}\sum_{n=1}^{N}{\left(\sqrt{{\tilde{B}}_{n}^{T}{\tilde{B}}_{n}}-\left\|B_{r}\right\|\right)}^{2}}$$

(15) $${\hat{e}}_{B}=\sqrt{\frac{1}{N}\sum_{n=1}^{N}{\left(\sqrt{{\hat{B}}_{n}^{T}{\hat{B}}_{n}}-\left\|B_{r}\right\|\right)}^{2}}$$

where $\left\{{\tilde{B}}_{n}\right\}$ and $\left\{{\hat{B}}_{n}\right\}$, n=1,2,⋯,N are the measurement data of the magnetic vector before and after calibrating the TAMCA, and $B_{r}$ is the true value of magnetic vector.

$a=\partial B_{x}/\partial x$, $b=\partial B_{y}/\partial y$, $d=(\partial B_{x}/\partial y+\partial B_{y}/\partial x)/2$, $e=\partial B_{z}/\partial y$, and $f=\partial B_{z}/\partial x$ are five independent components of the MGT measured by the TAMCA [25]. An ideal field used to calibrate the TAMCA is a uniform magnetic field and, therefore, the true values of a, b, d, e, and f are all zero. ${\tilde{e}}_{T}$ and ${\hat{e}}_{T}$ are the RMSEs of the MGT before and after calibration, whose expressions are as follows, respectively:

(16) $${\tilde{e}}_{T}=\sqrt{\frac{1}{N}\sum_{n=1}^{N}\left({\tilde{\delta }}_{an}^{2}+{\tilde{\delta }}_{bn}^{2}+{\tilde{\delta }}_{dn}^{2}+{\tilde{\delta }}_{en}^{2}+{\tilde{\delta }}_{fn}^{2}\right)}$$

(17) $${\hat{e}}_{T}=\sqrt{\frac{1}{N}\sum_{n=1}^{N}\left({\hat{\delta }}_{an}^{2}+{\hat{\delta }}_{bn}^{2}+{\hat{\delta }}_{dn}^{2}+{\hat{\delta }}_{en}^{2}+{\hat{\delta }}_{fn}^{2}\right)}$$

where $\left\{{\tilde{\delta }}_{an},{\tilde{\delta }}_{bn},{\tilde{\delta }}_{dn},{\tilde{\delta }}_{en},{\tilde{\delta }}_{fn}\right\}$ and $\left\{{\hat{\delta }}_{an},{\hat{\delta }}_{bn},{\hat{\delta }}_{dn},{\hat{\delta }}_{en},{\hat{\delta }}_{fn}\right\}$ are the errors of a, b, d, e, and f before and after calibrating the TAMCA, respectively.

### 3.2. Validation Using Magnetic Source Localization

The MIs calculated via the four individual TAMs are independent of the misalignment errors between the TAMs and of the spatial orientations of the TAMCA. Thus, unlike ${\hat{e}}_{T}$, ${\hat{e}}_{B}$ cannot be used to determine the accuracy of the vectors measured by the TAMCA. Moreover, the true value of the MI is often unknown for lots of in situ calibrations, and geomagnetic reference fields probably contain gradients caused by the magnetic material located nearby the experimental environments. This means that in certain geomagnetic field conditions, it is difficult to accurately and conveniently verify the effectiveness of TAMCA calibration through MI and MGT. Instead, the relative position between the AC magnetic source that generates magnetic anomaly field and the TAMCA is easier to obtain. Furthermore, the constrained Euler localization results of the AC magnetic source are both related to its magnetic vector and MGT [25]. Therefore, it is feasible to verify the effectiveness of calibrating the TAMCA using the constrained Euler localization of AC magnetic sources.

## 4. Calibration Algorithm Testing

### 4.1. Numerical Simulations

In numerical simulation experiments of calibration, $L_{x}$ and $L_{y}$ are all 0.5 m, the horizontal component H and vertical component Z of the geomagnetic field are 25.56 μT and 48.65 μT, and the magnetic inclination angle I and magnetic declination angle D are $62^{o}18^{\prime }$ and $-9^{o}55^{\prime }$, respectively. The noises of TAMs in the TAMCA are all zero-mean Gaussian random process, whose STD is denoted by $\sigma_{VM}$. The noise of scalar magnetometer measuring geo-MI is also a zero-mean Gaussian random process, whose STD is denoted by $\sigma_{SM}$. The error parameters of the TAMCA are shown in Table 1.

Two methods of rotating the TAMCA were used to simulate the generation of geomagnetic field data. One is to uniformly rotate around three axes of the TAMCA in turn, and the other is to randomly rotate around three axes of the TAMCA with a Gaussian distribution of Euler rotation angles. The simulation results of the relative calibration error obtained by the two methods are almost identical, so we adopted the random rotation in the simulation experiments. The parameters $\eta_{\delta S_{xi}}$, $\eta_{\delta S_{yi}}$, $\eta_{\delta S_{zi}}$, $\eta_{\theta_{i}}$, $\eta_{\varphi_{i}}$, $\eta_{\psi_{i}}$, $\eta_{B_{0xi}}$, $\eta_{B_{0yi}}$, $\eta_{B_{0zi}}$, $\eta_{\alpha_{j}}$, $\eta_{\beta_{j}}$, and $\eta_{\gamma_{j}}$ represent the relative errors of $\delta S_{xi}$, $\delta S_{yi}$, $\delta S_{zi}$, $\theta_{i}$, $\varphi_{i}$, $\psi_{i}$, $B_{0xi}$, $B_{0yi}$, $B_{0zi}$, $\alpha_{j}$, $\beta_{j}$, and $\gamma_{j}$, respectively. All numerical simulations use 50 Monte Carlo experiments to represent the noise distribution.

**Figure 4:**
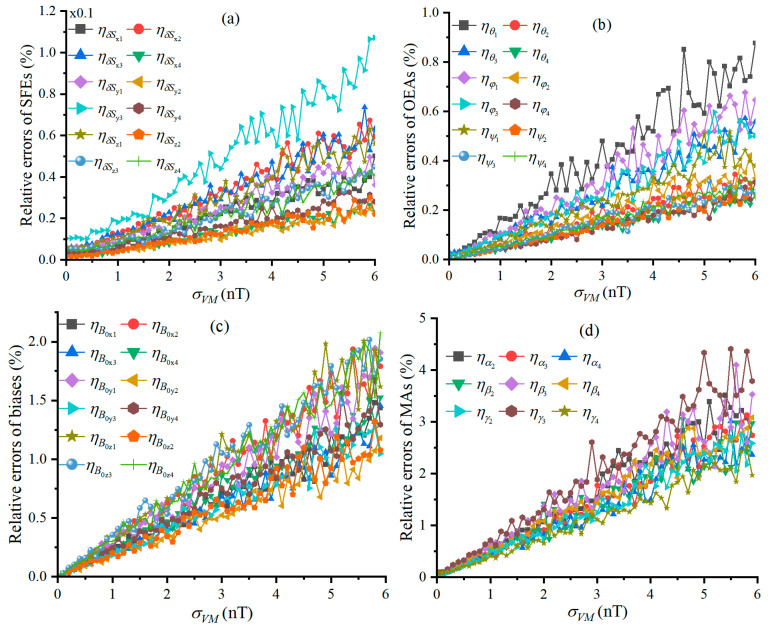
Relative error of calibrating TAMCA’s error parameters, varying with $\sigma_{VM}$, and Figures (**a**–**d**) are the curves of the relative errors of SFEs, OEAs, biases and MAs under different STDs of TAM’s noise.

The size N of the calibration dataset is 360 and a geo-MI of 54955.83 nT is provided by the scalar magnetometer. Figure 4 shows the plotted curves between the all relative errors and $\sigma_{VM}$, where these relative errors exhibit linear growth trends with the increase in $\sigma_{VM}$, and the fluctuation of this growth is evident when $\sigma_{VM}$ is greater than 1 nT.

**Figure 5:**
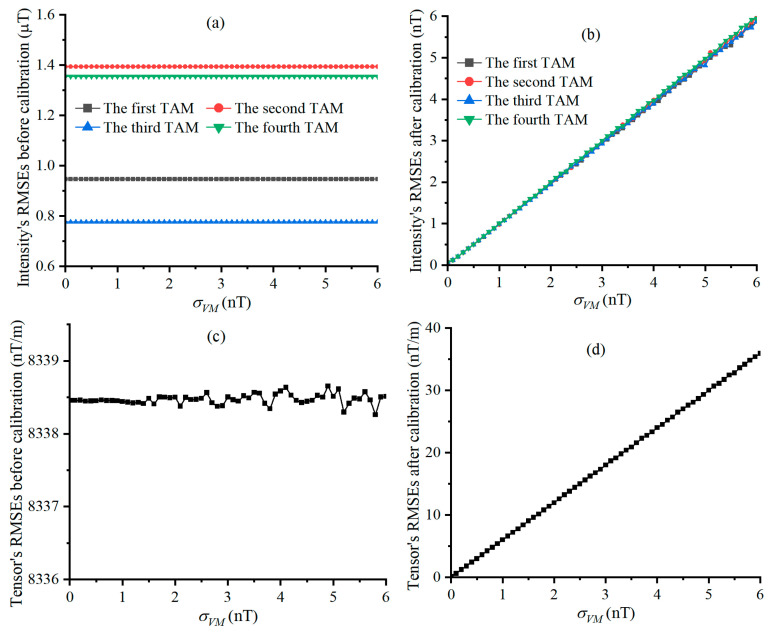
RMSEs of geo-MI and MGT, varying with $\sigma_{VM}$, Figures (**a**,**b**) are the curves of the intensity’s RMSEs before and after calibration, and Figures (**c**,**d**) are the Tensor’s RMSEs before and after calibration under different STDs of TAM’s noise.

${\tilde{e}}_{Bi}$ and ${\hat{e}}_{Bi}$ varying with $\sigma_{VM}$ are shown in Figure 5a and Figure 5b, and ${\tilde{e}}_{T}$ and ${\hat{e}}_{T}$ varying with $\sigma_{VM}$ are shown in Figure 5c and Figure 5d, respectively. ${\tilde{e}}_{Bi}$ and ${\tilde{e}}_{Ti}$ are almost unaffected by the increase in $\sigma_{VM}$, while ${\hat{e}}_{Bi}$ and ${\hat{e}}_{Ti}$ show positive and linear correlation with $\sigma_{VM}$. This is because the lossless scalar calibration greatly reduces the measurement error induced by the error parameters of the TAMCA, highlighting the effect of TAMs’ noise on the measurement accuracy of the geo-MI and MGT.

**Figure 6:**
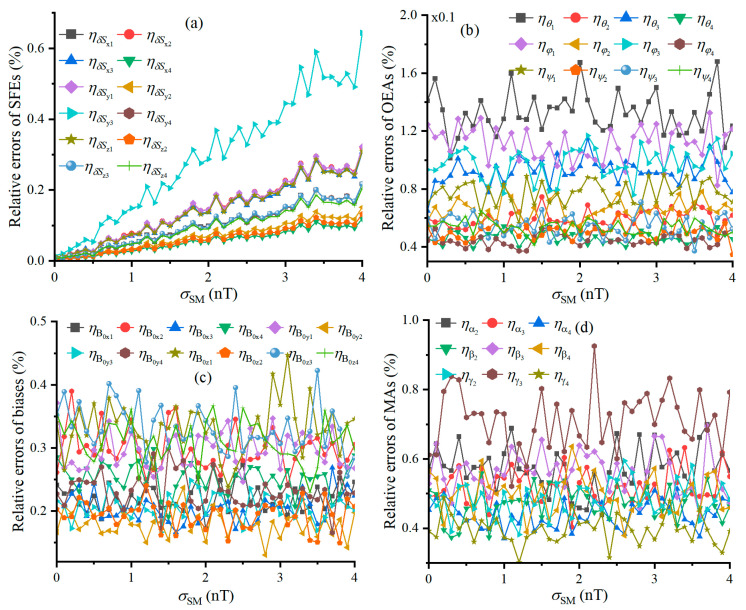
Relative errors of calibrating TAMCA’s error parameters, varying with $\sigma_{SM}$, and Figures (**a**–**d**) are the curves of the relative errors of SFEs, OEAs, biases and MAs under different STDs of TAM’s noise.

Similarly, the relationships between the relative errors and $\sigma_{SM}$ under $\sigma_{VM}=1\mathrm{nT}$ and N=360 are shown in Figure 6, where the relative errors of SFEs show linear growth trends with the increase in $\sigma_{SM}$ and the other relative errors remain almost invariant. This indicates that $\sigma_{SM}$ affects the calibration accuracy of $\delta S_{xi}$, $\delta S_{yi}$, and $\delta S_{zi}$, and it is the reverse for $\theta_{i}$, $\varphi_{i}$, $\psi_{i}$, $B_{0xi}$, $B_{0yi}$, $B_{0zi}$, $\alpha_{j}$, $\beta_{j}$, and $\gamma_{j}$.

**Figure 7:**
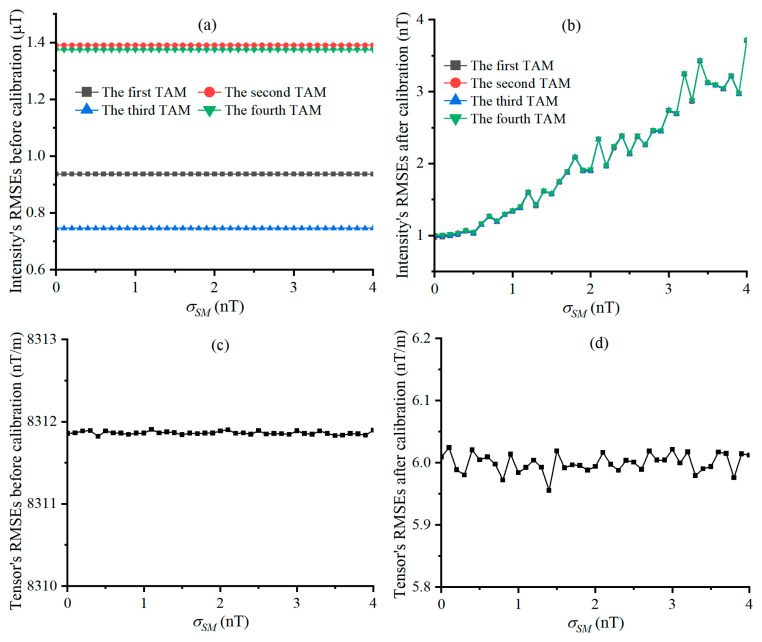
RMSEs of geo-MI and MGT, varying with $\sigma_{SM}$, Figures (**a**,**b**) are the curves of the intensity’s RMSEs before and after calibration, and Figures (**c**,**d**) are the Tensor’s RMSEs before and after calibration under different STDs of scalar magnetometer’s noise.

${\tilde{e}}_{Bi}$, ${\hat{e}}_{Bi}$, ${\tilde{e}}_{T}$, and ${\hat{e}}_{T}$ varying with $\sigma_{SM}$ are shown in Figure 7, respectively. Geo-MI and MGT measured by the TAMCA before calibration are not affected by $\sigma_{SM}$, and it is because the measured values of the geo-MI and MGT are calculated from the TAMs’ measurement data, unrelated to $\sigma_{SM}$. The comparison of Figure 7a with Figure 7b demonstrates that the calibrated TAMCA significantly reduces the measurement errors of the geo-MI, which tends to increase linearly with the increase in $\sigma_{SM}$, because it will lead to the increase in the measured reference geo-MI fluctuation. The comparison between Figure 7c,d proves that the calibrated TAMCA significantly reduces the measurement error of MGT, which is less affected by $\sigma_{SM}$ because the geo-MI is much greater than $\sigma_{SM}$.

**Figure 8:**
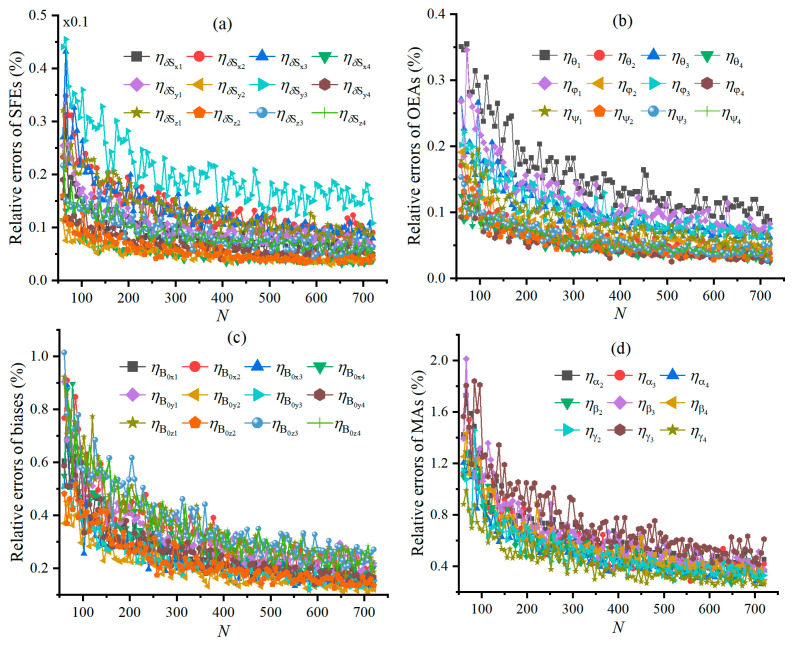
Relative error of calibrating TAMCA’s error parameters, varying with N, and Figures (**a**–**d**) are the curves of the relative errors of SFEs, OEAs, biases and MAs under different sizes of the calibration dataset.

Figure 8 shows the relative errors of calibrating TAMCA’s error parameters, as N changes when $\sigma_{VM}=1\mathrm{nT}$ and the scalar magnetometer provides a geo-MI of 54,955.83 nT. The relative errors all show decreasing trends with the increase in N; however, the calibration precision is less affected by N when it exceeds a certain threshold.

**Figure 9:**
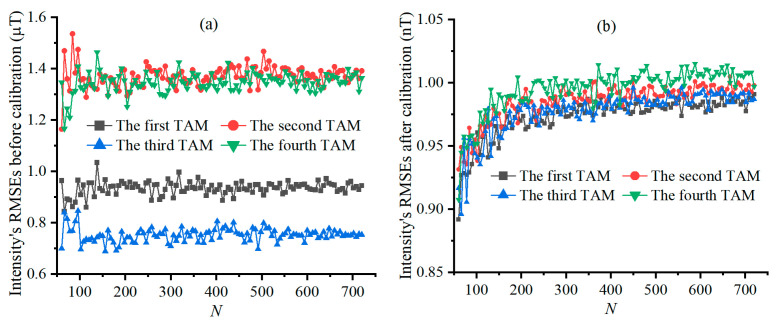

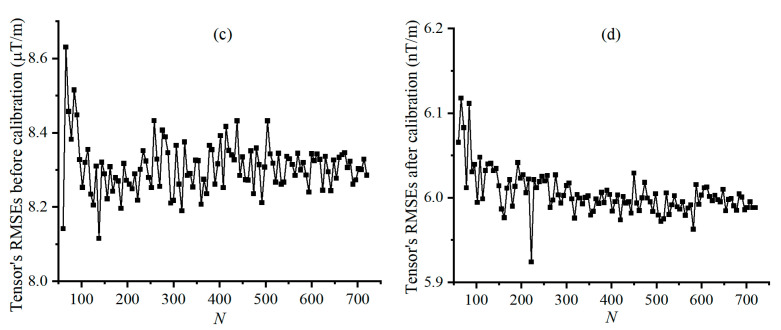
RMSEs of geo-MI and geo-MGT, varying with N, Figures (**a**,**b**) are the curves of the intensity’s RMSEs before and after calibration, and Figures (**c**,**d**) are the Tensor’s RMSEs before and after calibration under different sizes of the calibration dataset.

${\tilde{e}}_{Bi}$, ${\hat{e}}_{Bi}$, ${\tilde{e}}_{T}$, and ${\hat{e}}_{T}$ varying with N are shown in Figure 9, respectively. ${\tilde{e}}_{Bi}$ and ${\tilde{e}}_{T}$ are all nearly unaffected by N and yet their fluctuation reduces with the increase in N, as shown in Figure 9a,c. The reason is that the STD of the TAMs’ noise is much smaller than the measurement error of the geo-MI induced by the error parameters of the TAMCA. The comparison between Figure 9a,b demonstrates that the calibrated TAMCA significantly improves the measurement accuracy of the geo-MI, while it slightly increases and tends to stabilize with the increase in N. The comparison between Figure 9c,d reveals that the calibrated TAMCA significantly improves the measurement accuracy of the geo-MGT, while it slightly decreases and tends to stabilize with the increase in N. Their reasons are that the TAMs’ noise with an STD of 1 nT has an observable effect on the geo-MI and geo-MGT measured by the calibrated TAMCA. This also indicates that increasing N alone cannot further improve the calibration accuracy of the TAMCA when N reaches a certain threshold.

### 4.2. Experimental Tests

**Figure 10:**
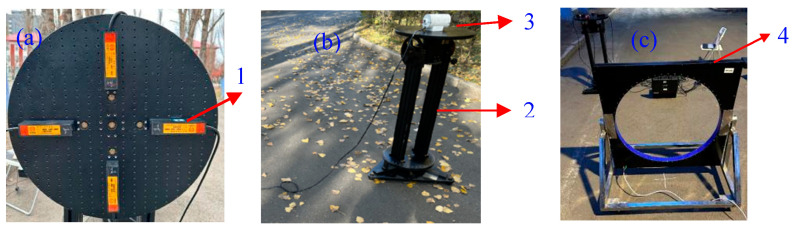
The experimental setup of testing the lossless scalar calibration algorithm: (**a**) is the FTAMCA placed on the table of the tri-axial non-magnetic turntable, (**b**) is the proton magnetometer placed on the table of the tri-axial non-magnetic turntable, and (**c**) is the energized circular coil; 1—FTAMCA (model: HSF133-2H3-AAB) with a noise level of no more than 15pTrms/Hz@1Hz and a measurement range of ±100 µT, 2—manual tri-axial non-magnetic turntable (model: HZT-500), 3—proton magnetometer probe (model: JHC-856T) with a resolution better than 0.1 nT, an accuracy better than 0.5 nT, and a measuring range from 0.01 µT to 100 µT, 4—the energized multi-turn circular coil.

**Figure 11:**
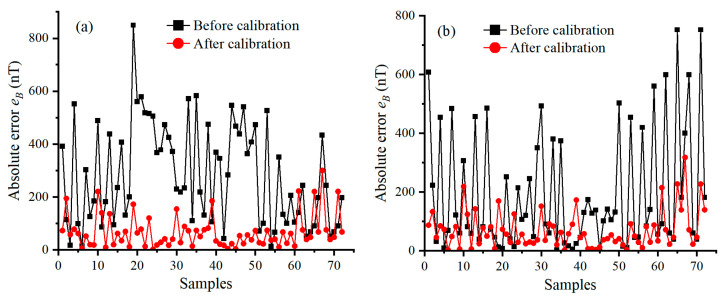

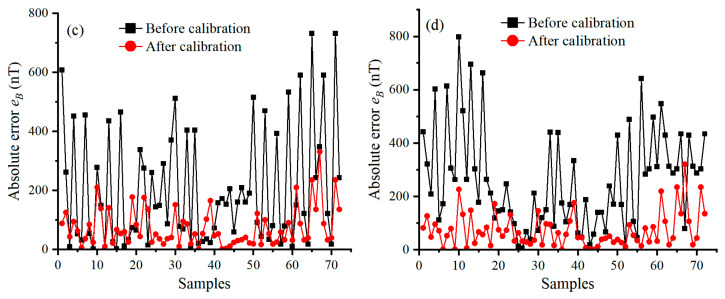
The absolute errors of geo-MI measured by the four FTAMs, Figure (**a**–**d**) represent respectively the absolute errors of geo-MI measured by the four FTAMs labeled by A, B, C and D before and after calibration.

The lossless scalar calibration algorithm is tested using a cross-shaped and symmetric array consisting of four FTAMs under an outdoor environment. The fluxgate TAMCA (FTAMCA) with the baseline length of 0.21 m is placed on the table of a tri-axial non-magnetic turntable, as shown in Figure 10a. Figure 10b illustrates the experimental setup of testing the lossless scalar calibration algorithm through the measurement of geo-MI, where the true geo-MI is provided by a proton magnetometer. Figure 11c shows an energized circular coil, which is regarded as an AC magnetic source to test the calibration algorithm through its constrained Euler localization.

The proton magnetometer with a sampling period of 5 s is used for in situ collection of the geo-MI. After smoothing and averaging the collected data, the geo-MI at the tri-axial non-magnetic turntable is 52185.0 nT. The initial angle of the turntable in the *x*-axis and *y*-axis is 0°, and the initial angle in the *z*-axis is −90°. Step 1: the turntable is rotated by one full circle around the *x*-axis with an interval of 60°. Step 2: the *y*-axis of the turntable is respectively adjusted to 60° and 120°, and then rotated around the *x*-axis following the way of the first step. Step 3: the *z*-axis of the turntable is adjusted to −60°, −30°, and 0° respectively; the rotation operations in both the first step and the second step are repeated. Therefore, the turntable has 72 sets of different orientations, and the corresponding dataset size is also 72. The duration of each set of data is approximately 20 s. The geomagnetic field of the calibration site of our university campus was measured by the proton magnetometer, and the STD of the geomagnetic field fluctuation was 8.26 nT, which could be regarded as magnetic noise or interference in the experiment.

Figure 11 illustrates the absolute errors of the geo-MI measured by four FTAMs of the FTAMCA before and after using the lossless scalar calibration algorithm, where black lines and red lines represent the absolute errors before calibration and after calibration, respectively. The absolute measurement errors of the geo-MI decrease from 339.2 nT, 277.2 nT, 283.2 nT, and 320.5 nT before calibration to 91.9 nT, 94.4 nT, 98.5 nT, and 97.1 nT after calibration, respectively. The results of calibrating the FTAMCA’s error parameters are presented in Table 2, and they are all within the range of the error parameters’ specification.

**Figure 12:**
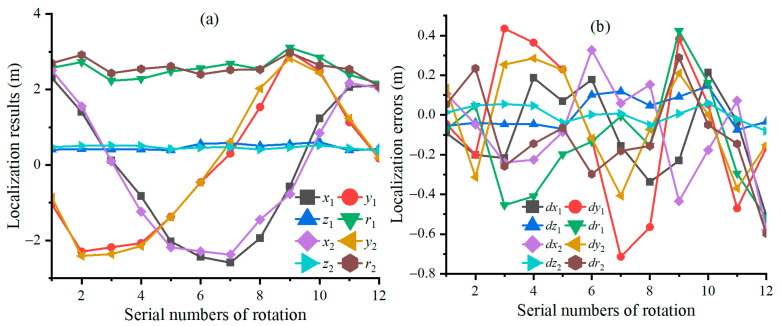
Localization results and their errors of the energized circular coil; the subscripts 1 and 2 denote, respectively, the values before and after calibrating the FTAMCA; (**a**,**b**) represent the localization results of the energized circular coil and the localization errors.

The power amplifier (model: IT7624) generates a sine voltage signal with peak of 10 V and frequency of 10 Hz, which are used to drive a multi-turn circular coil with inner and outer diameters of 0.82 m and 0.96 m, respectively. The number of turns in the circular coil can be calculated out according to its center magnetic field of 10Gs@3.3A. The initial angles of the turntable in the *x*, *y*, and *z* directions are all 0°. The position coordinates of the coil’s center relative to the FTAMCA’s center in the *x*-axis, *y*-axis, and *z*-axis are 2.40 m, −1.00 m, and 0.46 m, respectively. By rotating around the *z*-axis of the turntable with an interval of 30°, we can obtain 12 sets of magnetic field data of the energized circular coil measured by FTAMCA under different orientations. After these magnetic field data are processed using a band-pass digital filter, the constrained Euler localization algorithm is used to locate the circular coil, and the localization results and their errors are obtained as shown in Figure 12a and Figure 12b, respectively, where 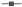, 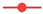, 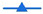, and 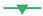 represent the results in the *x*-axis, *y*-axis, *z*-axis, and *r*-axis before calibration, and 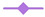, 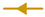, 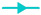, and 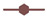 represent the results in the *x*-axis, *y*-axis, *z*-axis, and *r*-axis after calibration. The RMSEs of the localization in the *x*-axis, *y*-axis, *z*-axis, and *r*-axis are 0.2350 m, 0.3717 m, 0.0800 m, and 0.2924 m before calibration, and they are 0.2620 m, 0.2418 m, 0.0431 m, and 0.2516 m after calibration, respectively. Overall, the lossless scalar calibration of the FTAMCA improves the constrained Euler localization precision of the energized circular coil.

## 5. Conclusions

The four TAMs in the TAMCA are symmetrically arranged, with their intersection forming the measurement point of the TAMCA—a configuration that minimizes the principle-based measurement errors in the MGT compared to alternative designs. The paper proposes a lossless scalar calibration algorithm free from mathematical approximations, capable of tracking the reference MI fluctuations while precisely calibrating the SFEs, OEAs, and biases intrinsic to the four TAMs and the MAs between the TAMs. Simulation results of the relative errors calibrating the error parameters of the TAMCA demonstrate the calibration algorithm’s good performance under various measurement noise STDs and calibration dataset sizes, which can be reasonably explained. An experimental scheme based on constrained Euler localization is designed to validate the effectiveness of the lossless scalar calibration algorithm for TAMCAs. Validation experiments of calibrating the FTAMCA were conducted using a proton magnetometer and an energized circular coil, and the results demonstrated that the proposed algorithm significantly enhanced the measurement accuracy of MIs and improved the precision in determining the coil’s position coordinates relative to the FTAMCA. This underscores the calibration algorithm’s capability to mitigate systematic errors of multi-sensor magnetic measurement devices.

## Figures and Tables

**Table 1:** Error parameters of the TAMCA in numerical simulations.

Error Parameters	Unit	1st TAM	2nd TAM	3rd TAM	4th TAM
*δS_x_*		0.03	0.02	−0.02	−0.05
*δS_y_*		0.02	−0.04	0.01	0.03
*δS_z_*		−0.02	0.05	0.03	−0.03
*B*_0*x*_	nT	36	−27	38	31
*B*_0*y*_		22	35	−31	−28
*B*_0*z*_		−24	−41	24	−25
*θ*	°	0.14	−0.34	0.22	0.45
*φ*		−0.15	−0.26	−0.18	−0.38
*ψ*		0.21	0.35	0.31	0.32
*α*		0	−0.028	0.031	0.035
*β*		0	0.034	−0.028	−0.033
*γ*		0	−0.026	0.017	0.032

**Table 2:** The results of calibrating the FTAMCA’s error parameters.

Error Parameter	Unit	1st FTAM	2nd FTAM	3rd FTAM	4th FTAM
*δS_x_*		−0.0103	0.0062	0.0083	0.0023
*δS_y_*		0.0076	−0.0129	−0.0124	0.0140
*δS_z_*		0.0174	0.0038	−0.0004	−0.0143
*B*_0*x*_	nT	11.8467	−6.0092	−13.6844	−5.3428
*B*_0*y*_		26.1961	15.7185	10.3250	14.6912
*B*_0*z*_		−17.8786	10.6258	29.7575	2.9363
*θ*	°	0.0387	0.0174	0.0253	0.0165
*φ*		0.0646	0.0981	0.0861	0.0987
*ψ*		−0.0622	−0.0683	−0.0650	−0.0715
*α*		0	0.0164	0.0074	0.0127
*β*		0	0.0118	0.0117	0.0145
*γ*		0	−0.0093	−0.0155	−0.0080

## Data Availability

The data that support the findings of this study are available on request from the corresponding author.
